# Social Media Recruitment of Marginalized, Hard-to-Reach Populations: Development of Recruitment and Monitoring Guidelines

**DOI:** 10.2196/14886

**Published:** 2019-12-02

**Authors:** Jennifer Russomanno, Joanne G Patterson, Jennifer M Jabson Tree

**Affiliations:** 1 Department of Public Health University of Tennessee Knoxville, TN United States; 2 Arthur G James Cancer Hospital and Richard J Solove Research Institute/College of Public Health The Ohio State University Comprehensive Cancer Center Columbus, OH United States

**Keywords:** transgender, LGBTQ, TGNC, marginalized populations, cyberbullying, engagement, compassion fatigue, human subjects, research protections, adverse events

## Abstract

**Background:**

Social media can be a useful strategy for recruiting hard-to-reach, stigmatized populations into research studies; however, it may also introduce risks for participant and research team exposure to negative comments. Currently, there is no published formal social media recruitment and monitoring guidelines that specifically address harm reduction for social media recruitment of marginalized populations.

**Objective:**

The purpose of this research study was to investigate the utility, successes, challenges, and positive and negative consequences of using targeted Facebook advertisements as a strategy to recruit transgender and gender nonconforming (TGNC) people into a research study.

**Methods:**

TGNC adults living in the Southeast Unites States were recruited via targeted Facebook advertisements over two cycles in April and June 2017. During cycle 1, researchers only used inclusion terms to recruit the target population. During cycle 2, the social media recruitment and monitoring protocol and inclusion and exclusion terms were used.

**Results:**

The cycle 1 advertisement reached 8518 people and had 188 reactions, comments, and shares but produced cyberbullying, including discriminatory comments from Facebook members. Cycle 2 reached fewer people (6976) and received 166 reactions, comments, and shares but produced mostly positive comments.

**Conclusions:**

Researchers must consider potential harms of using targeted Facebook advertisements to recruit hard-to-reach and stigmatized populations. To minimize harm to participants and research staff, researchers must preemptively implement detailed social media recruitment and monitoring guidelines for monitoring and responding to negative feedback on targeted Facebook advertisements.

## Introduction

Between the years of 2008 and 2018, social media use among adults in the United States tripled from 21% to 69% [1]. Today, nearly 7 in 10 Americans use social media to connect with peers, engage with news content, and share information [1]. Across the available social media platforms, Facebook is the leading social network with 1.49 billion active members [2]. In the United States, 78% of adults aged 30 to 49 years and 64% of adults aged 50 to 64 years report using Facebook daily [3].

Given the increased popularity of social media platforms, social science researchers are using social media to recruit participants into health, medical, and psychosocial research studies [4-8]. Reliance on social media recruitment has grown as traditional recruitment methods (eg, flyers, newspaper advertisements, mailings, randomized digit dialing) continue to experience barriers to successful study recruitment, especially for hard-to-reach and stigmatized groups [8-10]. Researchers have successfully used Facebook to recruit a vast array of populations who experience stigma or discrimination due to their country of origin [11,12], race or ethnicity [13,14], sexual orientation [15,16], health behaviors [17,18] or mental health status [19,20]. Examples include Spanish-speaking Latino gay men [21], black women in HIV-prevalent urban areas [22], partnered gay men [23], long-term smokers [10], immigrants with limited English proficiency [24], and adults suffering from depressive symptoms [25].

Facebook may be a more effective recruitment tool than traditional methods for capturing marginalized groups. For example, Carter-Harris and colleagues [10] determined that Facebook advertising was a more effective strategy than newspaper advertisements for recruiting stigmatized lung cancer patients who were long-term smokers. Facebook advertising produced more participants than newspaper advertisements (311 vs 30) for substantially lower cost per participant ($1.51 vs $40.80). Researchers’ success in using Facebook as a recruitment tool for marginalized populations may be due to the varied levels of anonymity that social media affords users. Recruitment ads distributed via social media allow users to respond to online survey requests immediately and without requirement for in-person contact with the research team. Facebook users from stigmatized populations who view online recruitment ads may be more likely to participate in survey research that does not require direct contact with the research team, as is often required by traditional recruitment methods (ie, print advertisement providing an email or phone number for more information). For example, in Carter-Harris and colleagues’ study [10], lung cancer patients who feared smoking-related stigma were more successfully recruited in a completely online setting that allowed for greater privacy and anonymity during recruitment and participation.

Facebook users also control the visibility and authenticity of their online identities by including personally identifying details in profiles or restricting access to their profiles by other users via privacy settings. Consequently, when engaging with study recruitment ads distributed via Facebook, potential study participants can choose to remain anonymous or share their online identity by engaging with the recruitment advertisement (eg, by liking, commenting, or reposting). Accordingly, members of marginalized populations may feel more comfortable engaging with social media recruitment advertisements (eg, to ask questions or share study information) because they can choose profile and privacy settings to assert control over how much of their personal information is available to both study team members and other users.

Historically, transgender and gender nonconforming people (TGNC; individuals whose gender identity does not match their sex assigned at birth) [26] are underrepresented in peer-reviewed health-related literature [27,28]. One explanation is that most publicly available, population-based health surveillance surveys do not include gender identity measures [28,29]. Therefore, researchers wishing to document and describe the health of this population must recruit TGNC people into research studies via convenience samples in community-based spaces [30-32]. These spaces may be in-person via TGNC-inclusive organizations or groups or via online platforms, including social media [33].

While several studies have successfully used Facebook to recruit a wide range of hard-to-locate and/or stigmatized populations [4,5,10,22,23,34,35], there is little evidence describing the possible benefits and risks of using Facebook to recruit TGNC people. This is especially important because TGNC are at high risk of experiencing digital harassment, abuse, and cyberbullying online. In their study assessing cyberbullying among young adults worldwide, Myers and colleagues [36] concluded that transgender participants experienced digital harassment at a substantially higher frequency than cisgender (individuals whose gender identity matches their sex assigned at birth) males and females. Similarly, in a study assessing digital harassment and abuse among adults in England and Australia, almost two-thirds of transgender participants reported being threatened with physical harm by another person online and 60% reported experiencing digital harassment in the forms of offensive and degrading posts and direct messages about their gender identity and sexuality [37]. It is possible that TGNC participants who engage with research study advertisements distributed via Facebook may experience digital harassment, abuse, or cyberbullying in these forums; however, no evidence documents this phenomenon in the scholarly, peer-reviewed literature.

Using targeted Facebook advertisements is a relatively new recruitment method for engaging participants from highly stigmatized groups in research studies [38]. While several white papers and articles outline best practices and ethical considerations for researchers considering social media recruitment in general [6,39,40], no published guidelines exist in the peer-reviewed literature detailing safety and monitoring strategies for recruiting marginalized populations via social media. In the absence of evidence-based and best practice guidance, researchers cannot anticipate potential challenges or harms in social media recruitment of stigmatized populations or proactively build adequate safety and monitoring strategies into study protocols. Developing social media recruitment safety and monitoring guidelines require researchers who are using social media for study recruitment to be prepared to address risks of using social media including protecting participants and research staff and share methodological lessons learned to guide the development of safety and monitoring guidelines that can be applied to future recruitment-related processes.

The purpose of this research was to investigate the utility, successes, challenges, and positive and negative consequences of using targeted Facebook advertisements as a strategy to recruit TGNC people into a research study. We also sought to translate lessons learned from this study into recommendations for formal, duplicable guidelines (Social Media Recruitment Safety and Monitoring Guidelines) for researchers who are considering Facebook advertisements as a recruitment method for marginalized populations.

## Methods

### Ethics

The University of Tennessee institutional review board (IRB) approved all study procedures (UTK IRB-16-03275-XP).

### Recruitment

**Figure 1:**
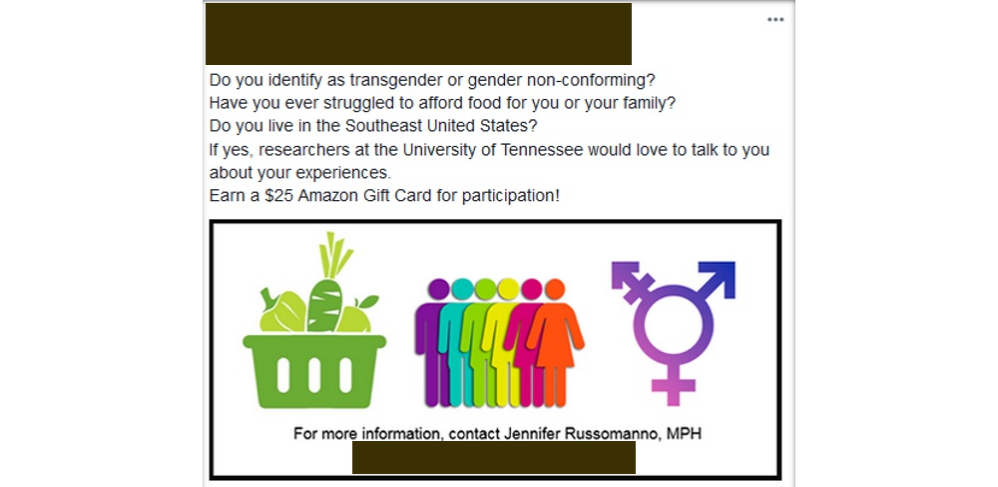
Targeted Facebook advertisement.

Participants for this study were recruited via targeted Facebook advertisements for an original study investigating experiences of food insecurity among TGNC people living in the Southeast United States [41]. Using a public Facebook page designed to represent a public health research lab at a state university, two successive recruitment cycles were conducted in April (Textbox 1) and June (Textbox 2) 2017. The same image and text (Figure 1) were used in both recruitment cycles, containing a brief introduction to the study and contact information for the study’s principal investigator (PI).

**Cycle 1 selection criteria.**

Inclusion criteria:

- Aged 18 to 50 years, male and female
- Interests: lesbian, gay, bisexual, transgender (LGBT)
- Locations: Alabama, Arkansas, Florida, Georgia, Kentucky, Louisiana, Mississippi, North Carolina, South Carolina, Tennessee, Virginia, and West Virginia
- Additional interests: Gay, Lesbian, Bisexual, Transgender, Straight Alliance; genderqueer; Lesbian, Gay, Bisexual, Transgender Community Center; National Center for Transgender Equality; trans women; transgender; transgender activism; Transgender Day of Remembrance; Transgender Law Center; transgenderism; Coming Out; Gay Pride; Gay Times; gender identity; homosexuality; Human Rights Campaign; LGBT community; LGBT network; LGBT social movements; Out Magazine; Pink (LGBT magazine); same-sex marriage; transgender youth

Exclusion criteria:

- None

**Cycle 2 selection criteria.**

Inclusion criteria:

- Aged 18 to 50 years, male and female
- Interests: lesbian, gay, bisexual, transgender (LGBT)
- Locations: Alabama, Arkansas, Florida, Georgia, Kentucky, Louisiana, Mississippi, North Carolina, South Carolina, Tennessee, Virginia, and West Virginia
- Additional interests: Gay, Lesbian, Bisexual, Transgender, Straight Alliance; genderqueer; Lesbian, Gay, Bisexual, Transgender Community Center; National Center for Transgender Equality; trans women; transgender; transgender activism; Transgender Day of Remembrance; Transgender Law Center; transgenderism

Exclusion criteria:

- Demographics > politics: US politics (conservative)
- Demographics > work > employers: Republican National Committee, Republican Party
- Interests > additional interests: Donald Trump, Guns & Ammo, Mike Pence, Paul Ryan, The Conservative

### Measures

Facebook monitors all forms of engagement in real time for the duration of any recruitment cycle and provides counts and summaries of each form of engagement at the cycle’s conclusion. We measured Facebook member engagement with each recruitment advertisement cycle by counting number and type of reactions, number of shares, and number and quality of comments. Likes, loves, and shares were counted as positive engagements. Angry, laughing, or sad reactions were counted as negative engagements. Laughter was considered a negative engagement because we interpreted laughing at the advertisement to be laughing at the recruitment content (food insecurity among those who identify as TGNC), which is not a positive affective response to a troubling public health issue.

We considered comments positive or negative based on their written content. A comment was considered negative if it included any derogatory language aimed at the target population (TGNC people) or study topic (food security), used profanity, or if the comment contained language that could be perceived as actual or potential threats of violence toward TGNC people. Comments were considered positive if they contained language that was supportive of TGNC people or the study topic.

Facebook business notifications are updates that Facebook sends to users reflecting any activity on Facebook advertisements or posts with which the user is associated [42]. Research team members were notified on their mobile phones each time there was engagement with the recruitment advertisement. Once the advertisement’s designated duration concluded, Facebook produced a summary of the advertisement’s overall results including the total number of Facebook members reached by the advertisement and the number of specific engagements on the advertisement. After each cycle was completed, the PI downloaded measures of engagement from Facebook for analysis.

### Analysis

We calculated summary and descriptive statistics using Facebook’s autogenerated engagement activity for both recruitment cycles. Counts of specific reactions, shares, and comments were tallied and summarized for each advertisement cycle. Individual advertisement cycle counts were then entered into an Excel spreadsheet (Microsoft Corp) and compared between cycles. The research team consistently and continuously monitored open-ended comments posted by Facebook members in reaction to each advertisement throughout each advertisement’s duration.

## Results

### Summary Statistics

**Figure 2:**
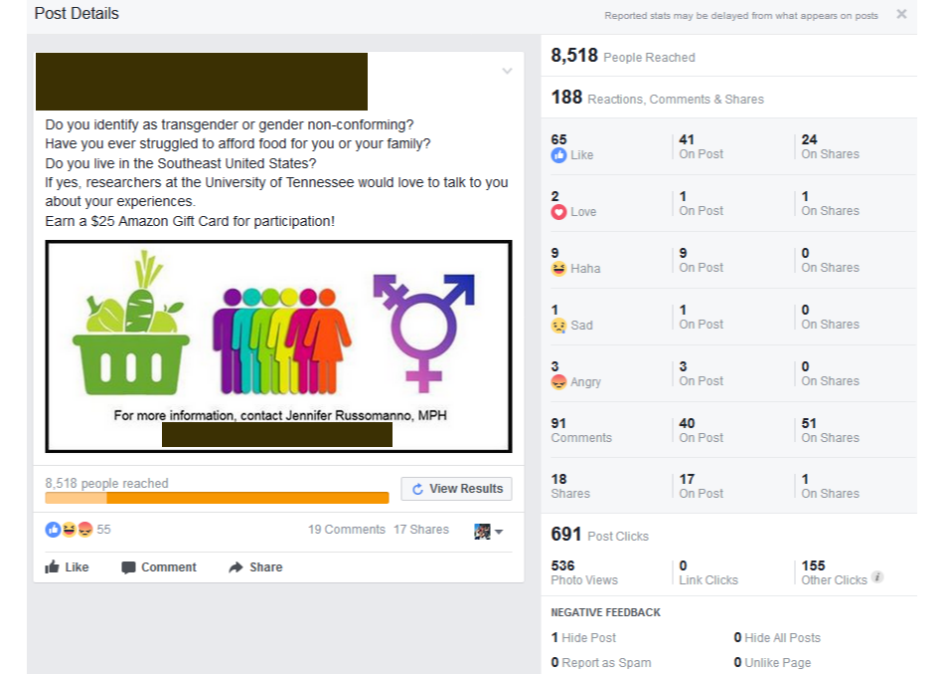
Cycle 1 advertisement Facebook summary report.

**Figure 3:**
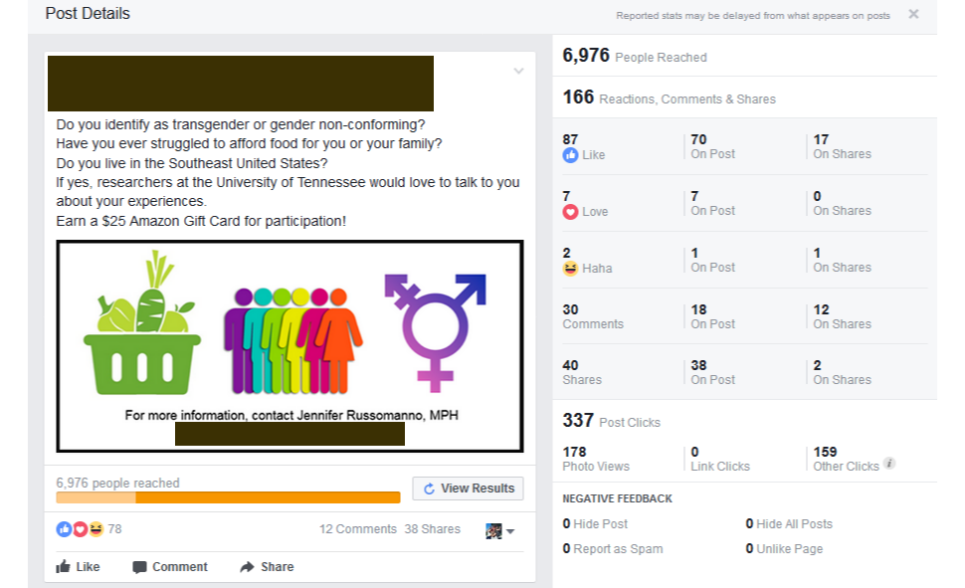
Cycle 2 advertisement Facebook summary report.

Table 1 summarizes engagement activity for both recruitment cycles. Figures 2 and 3 provide an overview of the cycle 1 and cycle 2 summary statistics autogenerated by Facebook at the conclusion of each cycle duration. We successfully recruited TGNC participants from both cycles. Seven participants were recruited from cycle 1. Seven participants were also recruited from cycle 2, with 3 additional people contacting the PI to participate after the study was closed to recruitment.

The cycle 1 advertisement reached 8518 Facebook members with 188 unique engagements (reactions, comments, and shares). Cycle 1 drew 85 positive engagements (65 likes, 2 loves, and 18 shares) and 12 negative engagements (3 angry reactions and 9 laughing reactions). Cycle 1 also drew several negative comments, which included derogatory, threatening, and discriminatory remarks about TGNC people. The cycle 2 advertisement reached 6976 Facebook members with 166 unique engagements. Cycle 2 drew 134 positive engagements (87 likes, 7 loves, and 40 shares) and only 2 negative engagements (laughing reactions). The cycle 2 advertisement received more positive feedback from Facebook members than cycle 1. The cycle 2 advertisement had a 40% increase in positive reactions (likes and loves: 67 in cycle 1 vs 94 in cycle 2) and a 122% increase in post shares by Facebook members compared with cycle 1 (18 in cycle 1 vs 40 in cycle 2). Compared with cycle 1, cycle 2 reached 178 more Facebook members on a daily basis due to the increase in advertisement post shares.

**Table 1:** Facebook engagements for recruitment cycles 1 and 2.

Interactions		Cycle 1 – 7 days (n=8518)	Cycle 2 – 5 days (n=6976)	Difference
Total engagement (reactions, comments, and shares)		188	166	–22
**Positive interactions**				
	Total likes	65	87	22
	Total loves	2	7	5
	Total shares	18	40	22
**Negative interactions**				
	Total haha	9	2	–7
	Total sad	1	0	–1
	Total angry	3	0	–3
**Other interactions**				
	Total comments	91	30	–61
	Total person reach per day	1217	1395	178

### Negative Consequences of Targeted Facebook Advertisements

During recruitment cycle 1, Facebook members negatively engaged with the advertisement by posting haha (n=9), angry (n=3), and sad (n=1) reactions. There were also several negative, cyberbullying comments made by Facebook members that were derogatory in nature, inflammatory, and potentially emotionally and mentally damaging to TGNC people. As soon as negative comments and cyberbullying began during cycle 1, research team members began continuously monitoring the advertisement for any potential negative comments, reactions, or private messages. Specifically, we assigned team members to respond to Facebook notifications during specific time periods, so that we could monitor posts 24 hours per day for the remaining duration of the recruitment cycle. Research team members immediately deleted negative comments made in response to advertisements.

When using targeted Facebook advertisements to recruit gay men into a 2015 research study, Mitchell and colleagues [23] received negative feedback to their advertisement in three forms: public comments posted on the Facebook advertisement and on the study’s public Facebook page, private messages sent to the Facebook study’s page, and voicemail. When addressing their experiences of cyberbullying during recruitment with Facebook representatives, Mitchell and colleagues [23] learned that any interests used as inclusion terms for targeted Facebook advertisements reach Facebook members who indicate either positive or negative views about a given interest. This means that Facebook members with negative views or opinions about a given interest could be inadvertently exposed to an advertisement. This unintended exposure creates a context in which Facebook members with negative views can engage with an advertisement, potentially creating a scenario in which these members make negative comments and engage in cyberbullying toward the intended study population [23].

**Figure 4:**
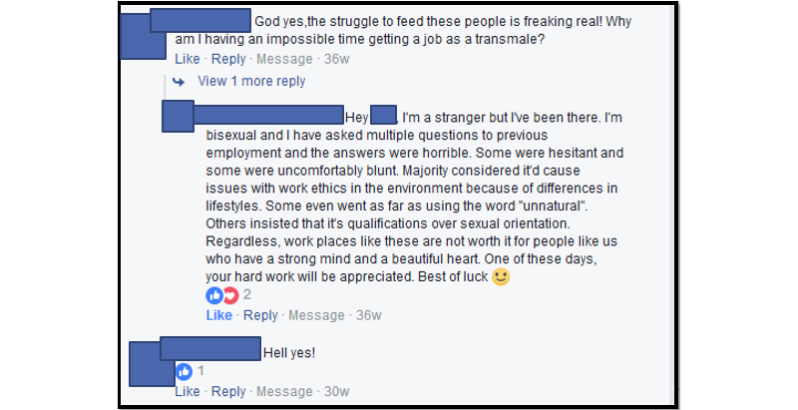
Sample supportive comment thread received during cycle 2.

During study recruitment cycle 1, the inadvertent exposure of the advertisement to Facebook members with negative views resulted in transphobic and discriminatory comments on the advertisement. After the cycle 1 experiences, we applied exclusion criteria similar to those set forth by Mitchell and colleagues [23] to the cycle 2 recruitment advertisement, and the second advertisement received more favorable and positive interactions from Facebook members. During cycle 2 recruitment, negative engagements were minimal, and there were only 2 haha reactions. Comments made by Facebook members during cycle 2 were positive and supportive of the TGNC community. Figure 4 shows an example of a positive comment thread received during cycle 2.

## Discussion

### Principal Findings

The purpose of this research study was to investigate the utility, successes, challenges, and positive and negative consequences of using targeted Facebook advertisements as a strategy to recruit TGNC people into a research study. TGNC people were quickly and effectively recruited into the study using Facebook; therefore, Facebook was determined to be a successful recruitment tool for the TGNC population. However, using Facebook to recruit TGNC people also produced unanticipated negative consequences for potential study participants, social media users viewing the advertisement in general (eg, TGNC people who were not potential participants), and research staff. During recruitment cycle 1, negative engagements in the form of degrading and derogatory comments were made by Facebook members on the advertisement’s post. The comments were discriminatory, which could have resulted in mental and emotional distress for potential study participants or other in-group (ie, TGNC people) or allied (eg, lesbian, gay, and bisexual people and other non-TGNC allies) social media users who viewed the advertisement. The receipt of negative and discriminatory comments is consistent with previous studies by Myers et al [36] and Powell et al [37] where study participants who identified as transgender reported high rates of digital harassment and abuse in online settings.

In addition to the potential damaging consequences for social media users, negative Facebook comments can also adversely affect research team members who manage and monitor the advertisement. For research team members who identify as members of or allies to the TGNC and lesbian, gay, bisexual, transgender, and queer (LGBTQ) communities, witnessing digital harassment and abuse can be especially damaging. In our study, the research team comprised three cisgender females who all identify as members of the LGBTQ community, and exposure to negative and stigmatizing comments toward other members of the LGBTQ/TGNC community resulted in secondary trauma for team members.

Secondary traumatic stress (STS), also known as compassion fatigue, experienced by research staff is not widely addressed in published literature. Qualitative scholars [43,44], those engaged in feminist social work [45], and those who conduct research with trauma counselors or therapists [46] discuss STS as a common emotional response to engaging with challenging or emotionally laden subjects or experiences. Researchers involved in the recruitment of stigmatized populations who witness and manage adverse events, including harassment and abuse, may experience similar instances of STS. For researchers engaged in difficult and challenging subject matter, STS can occur when team members are given the ability to see the world through their participants’ eyes [44]. In our study, team members were exposed daily to digital harassment and abuse faced by TGNC community members. Researchers who have a personal connection to the subject matter or who have experienced their own personal trauma are also at a high risk of experiencing STS [47]. To mitigate the potential effects of STS, we held weekly debrief sessions for all research team members to reflect and discuss emotional and psychological reactions arising from witnessing and responding to negative, degrading, and damaging Facebook advertisement comments.

### Social Media Recruitment and Monitoring Guidelines for Targeted Facebook Advertisements

With careful consideration and strategic planning, targeted Facebook advertisements can be a useful method for recruiting marginalized people into research studies. To the best of our knowledge, this is the first formal, published safety and monitoring guidelines for researchers using social media for this purpose. As our team experienced positive and negative repercussions while using Facebook advertisements for recruitment, we dynamically adjusted our monitoring strategies across recruitment periods to minimize harm for participants and research staff. Given our experience, we offer a specific guidelines for monitoring and responding to Facebook advertisements aimed at marginalized or stigmatized populations. We have also included recommendations for preparing and responding to participant and staff/research team exposure to negative comments or interactions on a Facebook advertisement.

### Monitoring

#### Defining Facebook Page Administration

Targeted Facebook advertisements are posted through publicly accessible Facebook pages associated with the study PI or research lab. To ensure adequate monitoring of the advertisement, at least two research team members should be assigned as administrators of the public page hosting the advertisement. Page administrators are able to define page and advertisement settings and receive automatic updates of posts to the public page or advertisement. We recommend that a research team use a shared decision-making process to assign page administrator roles, as page administrators work collaboratively to monitor and respond to comments made on the public page and study advertisements.

#### Notifications

To assure continuous monitoring, all page administrators should download the Facebook app to their mobile phones prior to beginning study recruitment. The administrators should monitor all notifications and interactions with the recruitment advertisement based on a predetermined schedule defined by the research team. Prior to launching the recruitment advertisement, the research team must determine how frequently (eg, hourly or daily) administrators should monitor the advertisement during active recruitment. A daily monitoring log should be established by research team members to ensure the advertisement has continuous monitoring throughout a cycle’s duration.

#### Recruitment Cycle Duration

Advertisements should be posted for, at maximum, 7 days per cycle to minimize burnout to research staff during recruitment while maximizing reach to the population of interest. Multiple recruitment cycles may be used until the desired sample size is achieved.

#### Inclusion and Exclusion Terms

To target advertisements to the study population of interest, researchers should use inclusion and exclusion terms based on study criteria. Inclusion terms include words, phrases, interests, or social/identity groups to which the advertisement applies. Exclusion terms include words, phrases, interests, or social/identity groups that may hold negative opinions of the target audience or research subject. Exclusion terms are included to guard against the inclusion of social media users who may engage in digital harassment and cyberbullying directed at the intended study population.

#### Public Page Settings and Moderation

**Figure 5:**
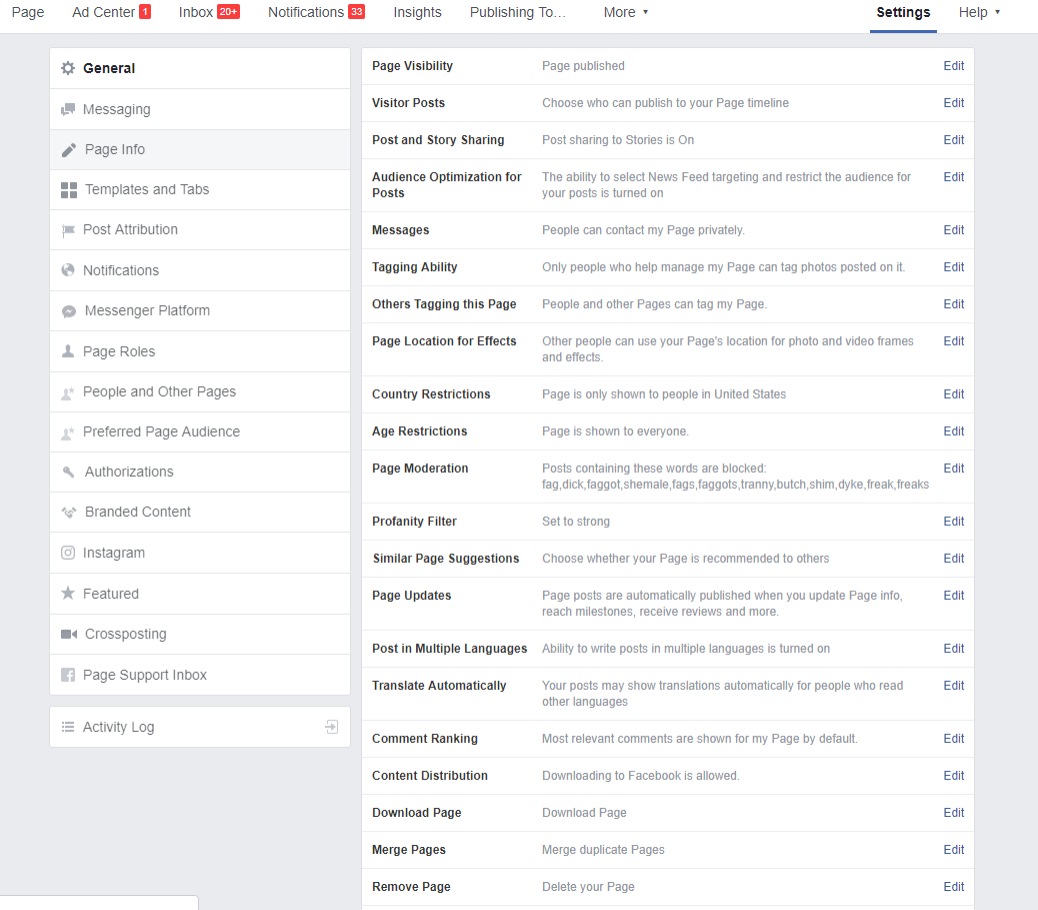
Public Facebook page settings.

Figure 5 outlines options that are available under the Settings tab on public Facebook pages or profiles. Researchers may restrict who can post directly to a public page by turning off the Visitor Posts feature. This ensures that only page administrators can post directly to the public site. If the study population of interest is US-based, researchers may restrict the country option to “US only” to ensure that only Facebook members residing in the United States can respond to the advertisement. This setting may be changed to direct advertisements to the researcher’s country of interest. While researchers cannot restrict social media users from commenting on the recruitment advertisement [48], the Facebook profanity filter can be set to high. This feature automatically restricts any comment that includes profanity from being posted to the recruitment advertisement. Additionally, under page moderation, the research team may block words or phrases deemed as derogatory toward the target population.

### Responding

While restricted and blocked comments are hidden to social media users, they can be seen by page administrators. As such, research team members have ample time to review and respond to profane or derogatory comments. For example, the research team may decide to unhide a comment if the content does not contain harassing or bullying behavior. However, in the case of threatening, negative, discriminatory, and/or bullying comments on recruitment advertisements, precautions must be taken. First, if not automatically blocked by Facebook via profanity or other filters, negative comments should be hidden by an authorized administrator. Researchers should continue to hide/block comments if the content constitutes digital harassment or cyberbullying. Research staff may also review blocked comments for patterns of repeated posts from troll users; these users may be blocked entirely from interacting with the public Facebook page (and associated advertisements) by page administrators. Facebook members responsible for negative or bullying comments may also be reported to Facebook for violating Facebook’s community standards policy [49].

Given that social media development is a dynamic process, we strongly encourage research teams to review Facebook’s updated privacy, page moderation, profanity, and reporting features prior to recruitment, while in the study planning phase. Reviewing these features and including them in a social media recruitment and monitoring guidelines proactively will help ensure that research teams are taking advantage of all automated protections offered by Facebook specific to page and advertisement moderation. Ultimately, the goal is to reduce burden on research staff in social media recruitment and monitoring while decreasing social media users’ exposure to negative comments and cyberbullying.

### Reporting

During the active recruitment and monitoring phase, all negative comments and interactions with social media and screenshots should be reported to the research PI by email within 24 hours. Screen shots should be saved by the PI in a secure, password-protected electronic location. The research team should reflect on their institution’s policies to determine if negative comments and interactions made on Facebook recruitment advertisements or the public page should be reported to their IRB. Human subjects research protections require that unanticipated problems that are unexpected, related, or possibly related to the research study and may place participants or others at greater risk of harm than was initially known or recognized be reported promptly to the IRB [50]. Specifically, US Department of Health and Human Services guidance notes that “Unanticipated problems that are serious adverse events should be reported to the IRB within 1 week of the investigator becoming aware of the event” [50]. However, due to the fast-paced nature of online recruitment, we recommend that any negative comments or interactions reported by study participants to the research team as harmful (eg, emotional or psychological harm, threatened physical harm) be reported by the PI to the IRB within 48 hours of the event.

All interactions with the advertisement are quantitatively captured by Facebook analytics, and summary reports should be downloaded by page administrators and recorded after each recruitment cycle. Each summary report should be saved and reviewed as necessary for tracking interactions with recruitment advertisements. The number of negative comments and their content (ie, specific statements) should be documented by the research PI.

### Dealing With Exposure to Negative Comments and Interactions

#### Research and Study Staff

We recommend that negative comments and interactions be processed and debriefed during frequent (ie, weekly) research team meetings to mitigate ST and compassion fatigue. This is especially important if study team members identify with the population of interest, as they may be at higher risk for experiencing STS. We also recommend that contact information for affordable and/or free and sliding scale psychological services be provided to research team members. For student research staff, this may include a mix of campus-based student psychological and mental health services and community-based resources. For employees, resources may include contact information for the employee assistance program and affordable community-based resources. It is important to consider the cultural background of research team members when providing community-specific resource lists. For example, for a study on LGBTQ or TGNC health wherein study team members also identify as LGBTQ, including LGBTQ-friendly resources is imperative.

#### General Public

It is possible that the general public may be exposed to negative comments and interactions on an advertisement for study recruitment before the comment can be identified and removed by study staff. We recommend that in the About section of the originating public social media page, page administrators list available support resources for individuals to access for support and coping with cyberbullying. These should be listed with appropriate contact information that is culturally specific to the region, topic, and/or target population and provides best possible accessibility (eg, a mix of national or regional hotlines, websites, and community-based resources as appropriate, including low or no-cost resources).

### Areas for Future Research on Recruitment-Related Processes

No formal guidelines existed for social media recruitment of marginalized populations. Guideline recommendations provided in this paper address this gap. However, these guidelines should continue to be tested and adapted as needed by researchers using Facebook to recruit marginalized or stigmatized populations. Additionally, researchers should consider adding qualitative interviews or focus groups to studies using social media recruitment methods to assess the experiences of recruited study participants.

### Conclusion

Facebook can be a useful tool when recruiting hard-to-reach, stigmatized populations. Targeted Facebook advertisements have the ability to reach large numbers of participants who otherwise may be hidden to research staff. However, for all the benefits that Facebook recruitment provides, there can be negative consequences of using this method. Creating detailed social media recruitment safety and monitoring guidelines in advance of using targeted Facebook advertisements may minimize and mitigate the risk to potential research participants and research team members.

## Abbreviations

IRB: institutional review board
LGBTQ: lesbian, gay, bisexual, transgender, and queer
PI: principal investigator
STS: secondary traumatic stress
TGNC: transgender and gender nonconforming
